# Seven Homologous Regions from a Novel *Bombyx mori* Nucleopolyhedrovirus (YT Strain) Isolate Enhance Early and Late Viral Promoters In Vitro and In Vivo

**DOI:** 10.3390/insects17080782

**Published:** 2026-07-28

**Authors:** Huifen Liu, Huiju Gao, Yu Xiu, Lifeng Hua, Lin Zhu, Guang Guo, Yaru Dong

**Affiliations:** 1 Shandong Institute of Sericulture, Shandong Academy of Agricultural Sciences, Yantai 264002, China; liuhuifen77@163.com (H.L.); weilan198106@163.com (Y.X.); hualifeng79@163.com (L.H.); zhulin_0517@163.com (L.Z.); ywk67@126.com (G.G.); 2 College of Forestry, Shandong Agricultural University, Taian 271018, China; ghj@sdau.edu.cn

**Keywords:** baculovirus, transient expression assay, enhancer, BmN cell line, silkworm

## Abstract

Homologous regions (*hr*s) are repetitive DNA sequences widely distributed in baculovirus genomes, and are known to function as transcriptional enhancers. In this study, we systematically characterized the seven *hr*s (*hr*1, *hr*2L, *hr*2R, *hr*3, *hr*4L, *hr*4R, and *hr*5) from a novel isolate of *Bombyx mori* nucleopolyhedrovirus (strain BmNPV-YT). The seven *hr*s range from 390 to 880 bp in length and consist of 1–7 repeat units of approximately 72 bp, each containing a 30 bp palindrome with an EcoRI site. Structural diversity among the *hr*s includes variable numbers of stem–loop motifs, major late transcription factor (MLTF) sites, cAMP response elements (CRE-like), and 12-O-tetradecanoylphorbol 13-acetate (TPA)-response elements (TRE-like). Functional assays using luciferase reporter plasmids revealed that all seven *hr*s function as potent transcriptional enhancers for both homologous and heterologous promoters in vitro and in vivo. In BmN cells and fifth-instar silkworm larvae, the *hr*s enhanced the homologous *ie1* promoter 11–1258-fold and 43–1545-fold, respectively, with *hr*2L showing the strongest activity (counts per minute: 4.82 × 10^8^ in cells; 7.08 × 10^7^ in larvae) and *hr*4R the weakest. Similarly, when driving the heterologous AcMNPV *ie1* promoter in Sf21 cells and larvae, enhancement ranged from 19- to 1023-fold and 13- to 449-fold, respectively, again with *hr*2L and *hr*3 showing maximal effects and *hr*4R minimal enhancement. Notably, all the *hr*s, except *hr*4R, also stimulated the homologous late *p10* promoter, with activation levels of up to 37.7-fold in vitro and 79.2-fold in vivo, significantly above baseline. However, *hr*4R showed negligible or no enhancement (0.84–1.05-fold). Among all the active *hr*s, *hr*5 emerged as the most potent enhancer of the *p10* promoter. Spearman’s correlation analyses demonstrated that enhancer efficiency for all three promoters (homologous *ie1*, heterologous *ie1*, and homologous *p10*) correlated significantly with the number of palindromic sequences, the combined count of CRE and TRE motifs, and the total number of characteristic sequences (r = 0.873–0.982, *p* < 0.05 or *p* < 0.01). Collectively, our findings clarify the structural features of *hr*s from the newly isolated strain BmNPV-YT, systematically identify their promoter preferences and host adaptability as transcriptional enhancers, and establish their structure–function relationships. These findings provide novel insights into the transcriptional regulation of baculoviruses and offer valuable tools for optimizing baculovirus expression vector systems.

## Simple Summary

The silkworm (*Bombyx mori*) is an important economic insect with great industrial value worldwide. *Bombyx mori* nucleopolyhedrovirus (BmNPV) is the most destructive pathogen of the silkworm, causing enormous economic losses to the sericulture industry. The strong pathogenicity of BmNPV is closely related to specific key regulatory regions in its genome, the homologous regions (*hr*s), which play two critical roles: as the “origin of replication” for the viral genome and as an “amplifier” promoting the efficient expression of other viral genes. In this study, we systematically characterized seven *hr*s in the genome of the newly isolated strain BmNPV-YT, revealing their divergence in the intensity of their activity, their cell and host specificity, and their regulatory effects on homologous and heterologous promoters. Notably, *hr*2L and *hr*3 were identified as highly efficient enhancers that boost early *ie1* promoter activity over 1000-fold in fifth-instar silkworm larvae. This study provides a solid experimental basis for clarifying the replication and transcriptional regulatory mechanisms of BmNPV and supports the rational optimization and application of the baculovirus expression vector system based on BmNPV.

## 1. Introduction

Baculoviruses are a family of invertebrate viruses characterized by large circular, double-stranded, covalently closed, supercoiled DNA genomes, ranging from 80 to over 188 kb, depending on the viral species [1]. Based on comprehensive genomic sequence analyses, baculoviruses are classified into four genera: *Alphabaculovirus* (lepidopteran-specific nucleopolyhedroviruses [NPV]), *Betabaculovirus* (lepidopteran-specific granuloviruses), *Gammabaculovirus* (hymenopteran-specific NPV), and *Deltabaculovirus* (dipteran-specific NPV) [2]. A defining genomic feature of nearly all baculoviruses studied to date is the presence of homologous regions (*hr*s). These regions are typically AT-rich, contain one or more copies of an imperfect palindromic sequence and various repeat motifs, and are dispersed at multiple locations within the genome. The first *hr*s were identified in the *Autographa californica* multiple nucleopolyhedrovirus (AcMNPV) genome [3]. AcMNPV contains nine *hr*s, each composed of 2–8 repeated units of approximately 70 bp, and all but *hr*4c feature a central 30 bp palindrome that usually contains an *Eco*RI restriction site [4,5].

Baculovirus *hr*s function as multifunctional *cis*-acting regulatory elements, playing two critical roles, as origins of DNA replication and potent transcriptional enhancers [6,7,8,9,10,11,12,13,14]. Their enhancer activities show broad specificity, effectively stimulating not only baculoviral early promoters, such as *ie1* [12], *ie2* [15], *ieN* [16], *39k* [17], *p35* [18], *p143* [19,20], and *cor* [21], but also late and very late promoters, including *ubi* [22], *gp64* [23,24], *vp39* [25], *p10* [26], *p6.9* [26], and *polh* [26,27]. Importantly, this enhancing capability extends across species boundaries. For instance, *hr*6 of *Hyphantria cunea* nucleopolyhedrovirus (HycuNPV) [15] and *hrs* 1–7 of *Spodoptera litura* nucleopolyhedrovirus II (*Splt*NPV-II) [28,29] can activate both homologous and heterologous promoters in various lepidopteran cell lines. However, accumulating evidence indicates the functional divergence among individual *hr*s within a single viral strain. For example, AcMNPV *hr*4a selectively enhances early promoters (*ie1* and *he65*) but exerts no effect on late promoters, such as *gp64*, *vp80*, *p6.9*, or *p10* [30]. Beyond viral promoters, *hr*s also significantly enhance the transcription from diverse non-baculoviral promoters, including host-derived promoters (e.g., the cytoplasmic actin gene [31], the larval serum protein gene (*lsp*) [24], *Bombyx mori* heat shock protein 70 (*hsp70*) [32], *B. mandarina hsp70* [32]), and heterologous promoters (e.g., *Drosophila hsp70* [15] and the human cytomegalovirus [CMV] promoter) in mammalian cells [4]. Moreover, recent multilocus manipulation studies have underscored the essential role of *hr*s in transcriptional regulation, demonstrating that AcMNPV *hr*1, *hr*2, and *hr*3 enhance the transcription of dozens of genes across adjacent 10–30 kb regions [14].

*Bombyx mori* nucleopolyhedrovirus (BmNPV), a major pathogen of the economically important silkworm (*B. mori*), causes significant losses to the sericulture industry. The BmNPV genome contains seven *hr*s distributed throughout its sequence, among which *hr*3 has been most extensively studied, with structural and functional variations observed across different viral strains. For example, *hr*3, reported by Lu (GenBank accession no. U77353), contains four 30 bp palindromes [31], whereas *hr*3 from strain T3 (GenBank accession no. L24902) contains six palindromes [33], and that from strain ZJ8 only three [34]. Another study has identified BmNPV-ZJ8 *hr*3 as a potent enhancer functioning both in vitro and in vivo. In Bm-5 cells, it enhanced the transcription from the homologous BmNPV *ie1* promoter 2421-fold; in BmN cells, it boosted the heterologous AcMNPV *ie1* promoter 6980-fold; and in silkworm larvae, *hr*3-mediated enhancement reached 1059-fold and 605-fold for homologous and heterologous *ie1* promoters, respectively [12].

Despite the extensive characterization of BmNPV *hr*3, there has been no systematic and comprehensive functional analysis of all seven *hr*s from a single BmNPV strain. In particular, the functional divergence among these seven *hr*s, their promoter preferences (especially for the early *ie1* and the very late *p10* promoter), and their host-specific regulatory patterns have not been fully clarified in different cellular and in vivo models (BmN cells, Sf21 cells, and silkworm larvae). Moreover, the structural determinants governing the enhancer potency of BmNPV *hr*s, such as the abundances of palindromic repeats and CRE and TRE motifs, have yet to be quantitatively established. These knowledge gaps collectively impede a complete understanding of the spatiotemporal transcriptional regulation in baculoviruses. In this study, we examined strain BmNPV-YT, a local isolate from our laboratory (GenBank accession no. PZ535956), which contains seven *hr*s, designated *hr*1, *hr*2L, *hr*2R, *hr*3, *hr*4L, *hr*4R, and *hr*5. To address the aforementioned gaps, we used transient expression assays to systematically analyze the transcriptional enhancement capabilities of these seven *hr*s on both homologous (BmNPV) and heterologous (AcMNPV) early *ie1* promoters in vitro (BmN and Sf21 cell lines) and in vivo (silkworm larvae). We also investigated the enhancing activities of these *hr*s on reporter gene expression driven by the homologous (BmNPV) very late *p10* gene promoter—a promoter widely used in the baculovirus–insect cell expression system (BEVS). We thus aimed to extend our fundamental understanding of baculoviral transcriptional regulation and provide optimized *cis*-regulatory tools for BEVS.

## 2. Materials and Methods

### 2.1. Materials

T4 DNA ligase, Platinum Pfx DNA polymerase, and Lipofectin 2000 Transfection Reagent were purchased from Invitrogen (Carlsbad, CA, USA). Taq DNA polymerase, restriction endonucleases (*Bam*HI, *Sal*I, *Kpn*I, *Hin*dIII, etc.), a DNA purification kit, a luciferase assay kit, the pRL-CMV vector (used for internal control transfections), and the pGL3-Basic vector (used as the blank control) were obtained from Promega (Madison, MI, USA). The pEASY-T3 Cloning Kit was acquired from TransGen Biotech (Beijing, China). *Escherichia coli* strain DH10B was maintained in our laboratory. The reporter plasmids pBm*ie1*P/*luc* and pAc*ie1*P/*luc*, containing the luciferase (*luc*) gene driven by the BmNPV *ie1* promoter and the AcMNPV *ie1* promoter, respectively, were constructed in our previous study [29]. All primer sequences used for PCR amplification are listed in Table 1.

### 2.2. Virus, Cell Lines, and Silkworm Larvae

Strain BmNPV-YT was isolated and is maintained in our laboratory. Both BmN and Sf21 cell lines were propagated at 27 °C in TC-100 insect medium supplemented with 10% heat-inactivated fetal bovine serum (Invitrogen; inactivation: 56 °C, 30 min). Cell culture procedures followed the methods established by Summers and Smith [35]. Silkworms (strain Jingsong × Haoyue) were purchased from Shandong Guangtong Silkworm Egg Co., Ltd. (Qingzhou, China). Larvae were reared on fresh mulberry leaves under standard conditions throughout larval development [36].

### 2.3. Construction of Transient Expression Plasmids

**Figure 1:**
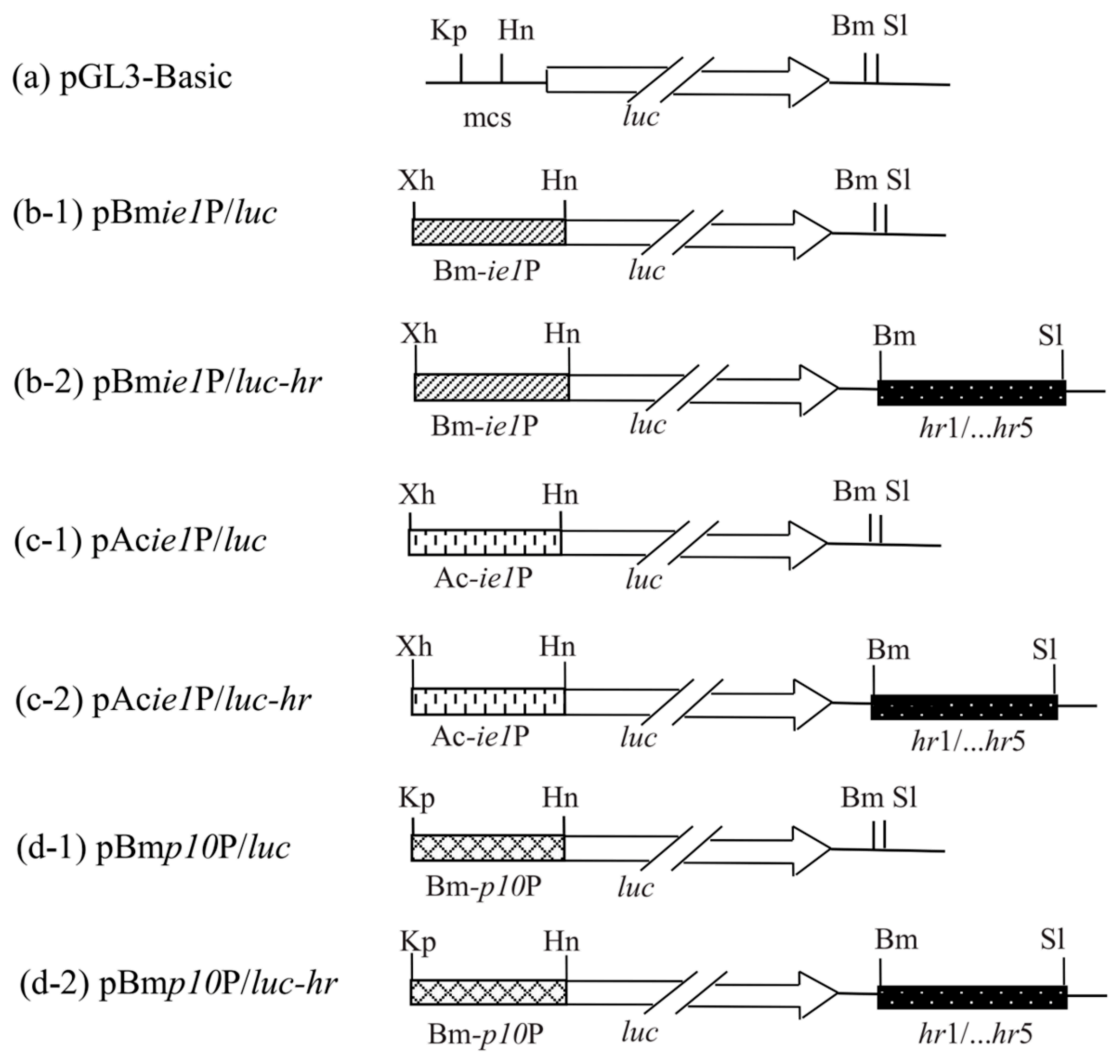
Diagrams of reporter vector constructs. Schematic representations of the reporter vectors used to assess the enhancement activities of seven *hr*s. (**a**) Schematic of the promoter-less empty pGL3-Basic vector containing the luciferase reporter gene (*luc*) and multiple cloning site (MCS). (**b-1**,**b-2**) Bm-*ie1* promoter-based reporter vectors. (**b-1**) pBm*ie1*P/luc, carrying the Bm-*ie1* promoter upstream of the *luc* gene; (**b-2**) pBm*ie1*P/luc-*hr*, containing the Bm-*ie1* promoter and the Bm-*hr*1–*hr*5 fragment. (**c-1**,**c-2**) Ac-*ie1* promoter-based reporter vectors. (**c-1**) pAc*ie1*P/luc, carrying the Ac-*ie1* promoter upstream of the *luc* gene; (**c-2**) pAc*ie1*P/luc-*hr*, containing the Ac-*ie1* promoter and the Bm-*hr*1–*hr*5 fragment. (**d-1**,**d-2**) Bm-*p10* promoter-based reporter vectors. (**d-1**) pBm*p10*P/luc, carrying the Bm-*p10* promoter upstream of the *luc* gene; (**d-2**) pBm*p10*P/luc-*hr*, containing the Bm-*p10* promoter and the Bm-*hr*1–*hr*5 fragment. The luciferase reporter gene (*luc*) is indicated by arrows. The *Bm-ie1*, *p10*, and *Ac-ie1* promoters and *Bm-hr1–hr5* enhancers are distinctively shown and indicated. Multiple cloning sites (mcs) are represented by multiple vertical bars. Key restriction enzyme sites are abbreviated as follows: Kp, *Kpn*I; Hn, *Hin*dIII; Bm, *Bam*HI; Sl, *Sal*I; Xh, *Xho*I.

**Figure 2:**
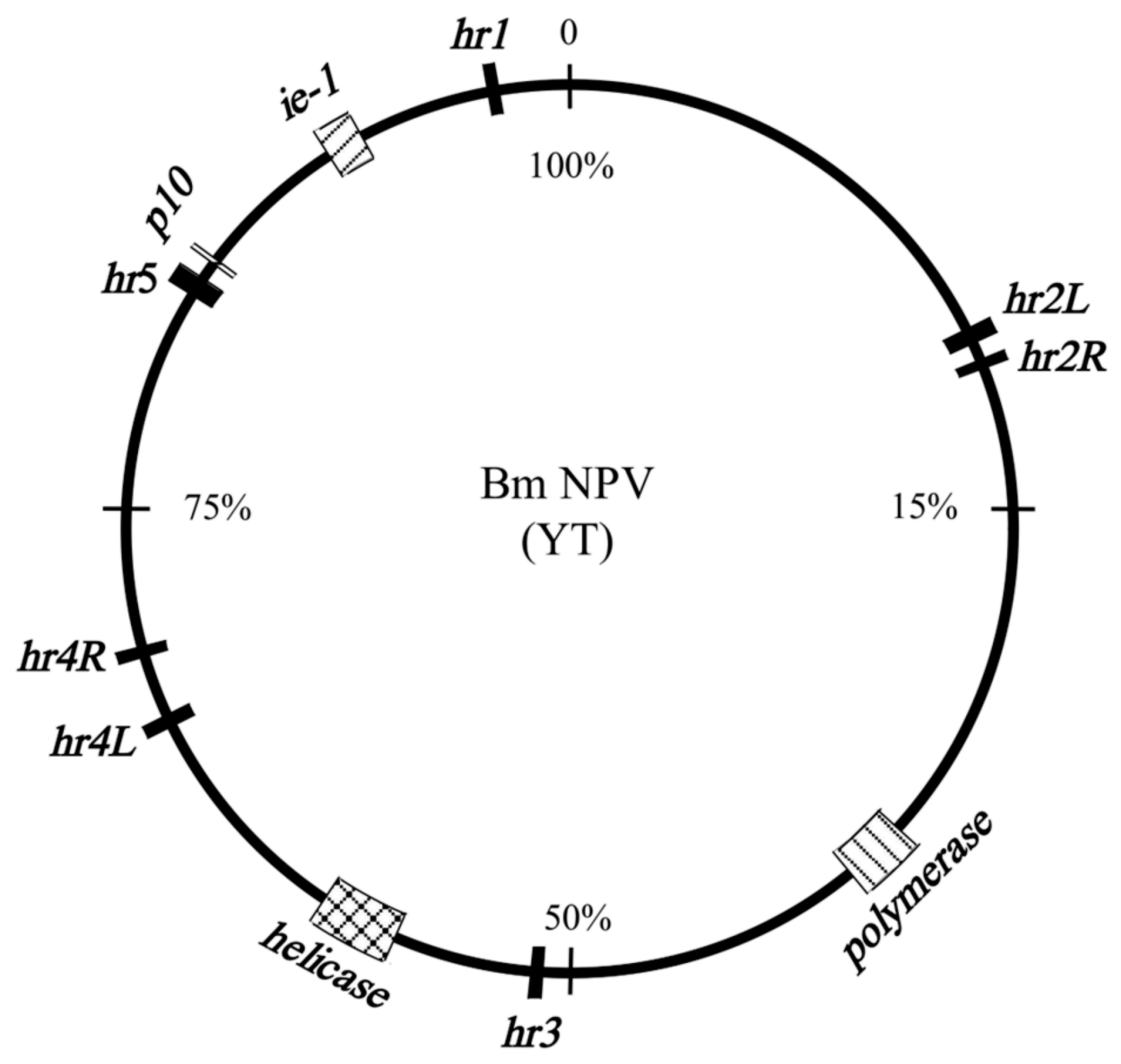
Positions and sequence information of seven *hr*s in BmNPV-YT genome. Locations of the different *hr*s in the BmNPV-YT genome are indicated. *hr*1: 461 bp (nt 123481–123941), *hr*2L: 875 bp (nt 22448–23322), *hr*2R: 395 bp (nt 23989–24383), *hr*3: 561 bp (nt 64765–65325), *hr*4L: 552 bp (nt 86006–86557), *hr*4R: 467 bp (nt 89436–89902), and *hr*5: 839bp (nt 106617–107455).

Seven *hr* elements (*hr*1, *hr*2L, *hr*2R, *hr*3, *hr*4L, *hr*4R, and *hr*5) and the *p10* promoter fragment (325 bp) were amplified with PCR using the BmNPV-YT genomic DNA as a template (Table 1). Each *hr* fragment was individually subcloned into the reporter vectors pBm*ie1*P/luc and pAc*ie1*P/luc, generating two plasmid series: (1) the pBm*ie1*P/luc series: pBm*ie1*P/luc-*hr*1, -*hr*2L, -*hr*2R, -*hr*3, -*hr*4L, -*hr*4R, and -*hr*5; and (2) the pAc*ie1*P/luc series: pAc*ie1*P/luc-*hr*1, -*hr*2L, -*hr*2R, -*hr*3, -*hr*4L, -*hr*4R, and -*hr*5 (Figure 1b-2,c-2). Subsequently, the *ie1* promoter was replaced with the *p10* promoter in pBm*ie1*P/luc and all its *hr*-containing derivatives (pBm*ie1*P/luc-*hr*1 to *hr*5). This yielded functional plasmids designated pBm*p10*P/luc, pBm*p10*P/luc-*hr*1, -*hr*2L, -*hr*2R, -*hr*3, -*hr*4L, -*hr*4R, and -*hr*5, respectively (Figure 1d-2). All the constructs were verified by direct sequencing (Sanger sequencing, ABI 3730XL, Sangon Biotech, Shanghai, China) to confirm the correct insertion orientation and sequence integrity.

### 2.4. Transfection of Insect Cells and Fifth-Instar Silkworm Larvae

Routine cell culture was performed according to Summers and Smith [35]. Cell transfection was performed with a previously described protocol [12]. Briefly, BmN and Sf21 cells were cotransfected with 50 μL of transfection solution containing 1 µg of the reporter plasmid DNA, 0.2 µg of the internal control plasmid DNA, and 3 μL of Lipofectin Transfection Reagent. Cells transfected with the empty pGL3-Basic vector were used as the blank control. For viral infection, cells were incubated with virus at a multiplicity of infection (MOI) of 3 in serum-free medium for 1 h, after which the medium was replaced with fresh complete medium. All experiments were performed with three independent biological replicates.

Fifth-instar silkworm larvae were transfected using the method of Chen [12]. At 48 h after molting, the hemolymph of each fifth-instar larva was injected with 20 μL of transfection solution containing 1 µg of the reporter plasmid DNA, 0.2 µg of the internal control plasmid DNA, and 2 μL of Lipofectin Transfection Reagent. For viral infection, each larva was injected with wild-type BmNPV-YT (5.0 × 10^5^ plaque-forming units) 6 h before transfection. Each treatment was applied to at least three separate groups (with 10 larvae of similar bodyweight per group) and was repeated three times.

### 2.5. Luciferase Activity Assay

The *hr* activities were assayed with the Dual-Luciferase Reporter Assay System (Promega, Madison, MI, USA) at 48 h posttransfection (hpt), and the transfected BmN cells, Sf21 cells, and larval hemolymph cells were harvested by centrifugation (5000× *g*, 5 min, 4 °C). The cell pellets were washed twice with phosphate-buffered saline (PBS) and lysed according to the manufacturer’s protocol. The lysate protein concentration was determined with the Bradford method, as previously described [37]. Luciferase activity was measured with a GloMax 20/20 Luminometer (Promega) [28] and expressed as counts per minute (CPM) in 15 s. The pRL-CMV plasmid was used to normalize the luciferase activity in each extract, according to the manufacturer’s instructions. All data were collected from triplicate assays of three independent transfections.

### 2.6. Data Analysis

The DNAMAN10 software (https://www.dnaman.net, accessed on 1 June 2026) was used to generate a schematic diagram of the distribution of *hr*s in the BmNPV-YT genome and to analyze sequence homologies. To analyze the correlation between the enhancer activity of each *hr* fragment and the abundance of CRE and TRE motifs or palindromic sequences, Spearman’s rank correlation coefficient was calculated with SPSS Statistics 17.0 (SPSS Inc., Chicago, IL, USA). Statistical significance was defined as * *p* < 0.05, ** *p* < 0.01, or *** *p* < 0.001.

## 3. Results

### 3.1. Characteristic Structure of the Seven Homologous Regions in Strain BmNPV-YT

The seven *hr*s are dispersed throughout the BmNPV-YT genome, which comprises 127,170 bp, with individual *hr* lengths ranging from approximately 390 to 880 bp (Figure 2). Each *hr* is composed of one (*hr*4R) to seven (*hr*2L and *hr*5) repeat units of around 72 bp, each containing a central 30 bp palindromic core that typically contains an *Eco*RI restriction site. Notably, *hr*4R contains only one repeat unit, which has a single-nucleotide mutation within its *Eco*RI site. These *hr*s contain diverse conserved regulatory motifs, including stem–loop structures, major late transcription factor (MLTF) sites, cAMP-response elements (CRE-like) and 12-O-tetradecanoylphorbol 13-acetate (TPA)-response elements (TRE-like), with distinct copy numbers among the different *hr*s (Table 2). The repetitive sequences share 72–90% nucleotide identity across the entire combined *hr*1–*hr*5 regions, whereas repeats within the same individual *hr* display approximately 90% sequence identity. Multiple sequence alignments were performed between the *hr* regions of the BmNPV YT strain and those of the classical BmNPV T3 and ZJ strains, using the reference sequences (GenBank accession numbers: L24899–L24905, JQ991008.1). The consensus sequences of YT *hr*s share approximately 80% identity with the corresponding *hr* regions of the T3 and ZJ strains.

### 3.2. Stimulatory Efficiency of the Seven hrs on the Homologous ie1 Promoter In Vitro and In Vivo

To clarify the regulatory roles of the seven *hr*s of BmNPV-YT on its homologous *ie1* promoter, we transfected reporter plasmids carrying the luciferase gene (*luc*) driven by the *ie1* promoter both in vitro (in BmN cells) and in vivo (in fifth-instar silkworm larvae). The enhancer activities of these *hr*s are summarized in Table 3 and Table 4, respectively. All seven *hr*s showed enhancer activity in both systems, although the magnitudes of enhancement varied significantly.

In BmN cells, the seven *hr*s enhanced transcription from the homologous *ie1* promoter 11–1258-fold. In fifth-instar silkworm larvae, the enhancement ranged from 43- to 1545-fold, representing a markedly stronger effect than was observed in vitro. Among the seven *hr*s, *hr*2L displayed the strongest enhancer activity, yielding CPM values of 4.82 × 10^8^ in BmN cells and 7.08 × 10^7^ in larvae. *hr*3 ranked second, mediating 721-fold enhancement in cells and 1026-fold enhancement in larvae. In contrast, *hr*4R showed the weakest effect in both assays, with only 11-fold enhancement (CPM: 4.29 × 10^6^) in vitro and 43-fold enhancement (CPM: 1.98 × 10^6^) in vivo.

### 3.3. Stimulatory Efficiency of the Seven hrs on the Heterologous ie1 Promoter In Vitro and In Vivo

To determine whether the seven *hr*s enhanced the activity of the heterologous AcMNPV *ie1* promoter, we individually transfected Sf21 cells (in vitro) and fifth-instar silkworm larvae (in vivo) with reporter plasmids containing the *luc* gene driven by this reporter. As shown in Table 5 and Table 6, luciferase activity was detected in all transfections when the heterologous *ie1* promoter was stimulated by any of the seven *hr*s. In Sf21 cells, the transcriptional activity of the heterologous *ie1* promoter was enhanced 19- to 1023- fold in the presence of the *hr*s. Similarly, in silkworm larvae, a 13–449-fold increase in activation was observed. These results indicated that *hr*2L and *hr*3 exerted strong enhancer effects on the heterologous early promoter both in vitro (CPM: *hr*2L, 5.58 × 10^8^; *hr*3, 5.06 × 10^8^) and in vivo (CPM: *hr*2L, 2.42 × 10^7^; *hr*3, 1.87 × 10^7^). In contrast, *hr*4R displayed the weakest enhancement of the heterologous *ie1* promoter—a pattern consistent with its effect on the homologous *ie1* promoter. The remaining four *hr*s (*hr*1, *hr*2R, *hr*4L, and *hr*5) displayed intermediate enhancement capabilities for both promoters.

### 3.4. Stimulatory Efficiency of the Seven hrs on the Homologous p10 Promoter In Vitro and In Vivo

Previous studies have shown that certain *hr*s can function not only as activators of viral early promoters but also as enhancers of the gene expression driven by viral late promoters. To determine whether the seven *hr*s identified in BmNPV-YT exert enhancer activity on a late promoter, we generated a series of reporter plasmids. Each plasmid contained the *luc* gene under the control of the homologous BmNPV-YT *p10* late promoter, together with one of the seven *hr* sequences. For the cell-based assay, BmN cells were infected with the wild-type virus and then transfected with the individual reporter plasmids. For the in vivo assay, fifth-instar larvae were injected with the virus, followed by transfection with the reporter plasmids. Cells and hemolymph were harvested at 48 hpt, and luciferase activity was measured to assess the enhancer function.

**Figure 3:**
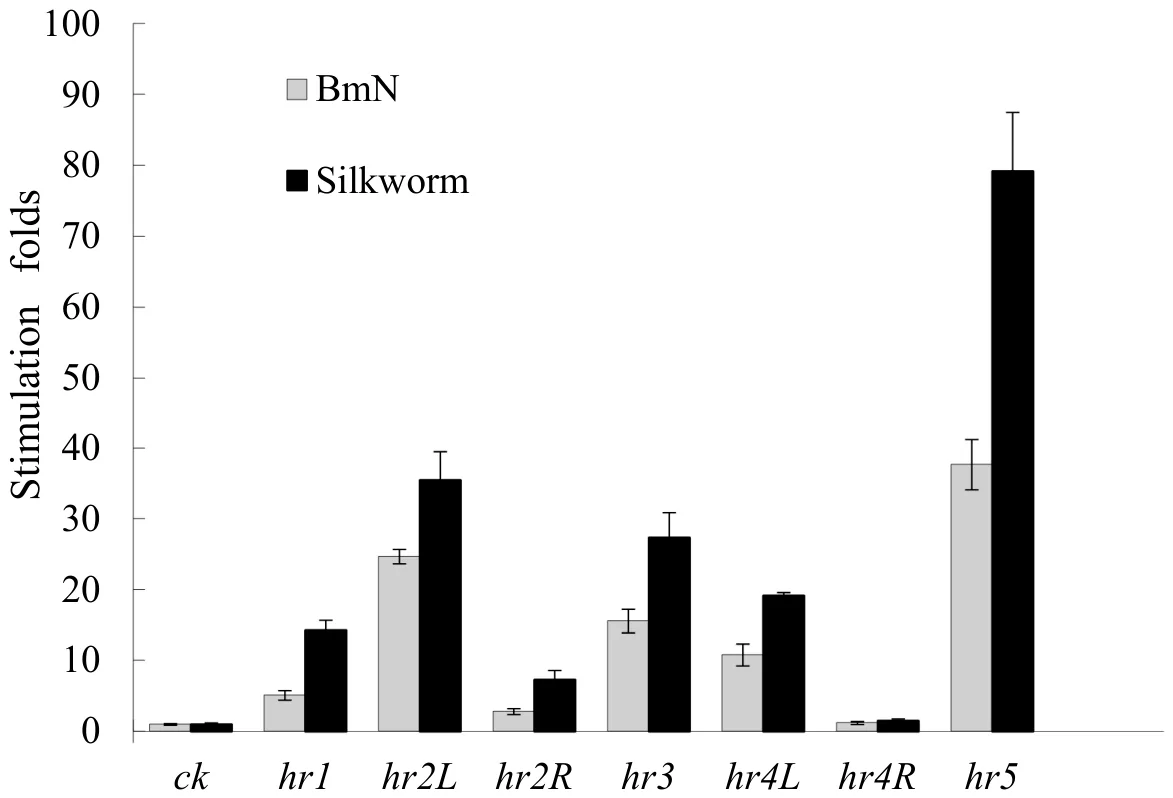
Enhancement of *p10* promoter activity by seven *hr*s in vitro and in vivo. Gray histograms indicate relative luciferase activity of each *hr-*containing construct compared with that of the *hr*-less pBm*p10*P/luc construct in BmN cells. Black histograms show the corresponding activities in fifth-instar silkworm larvae. Error bars represent standard deviations from three independent replicates.

The enhancer effects of the seven *hr*s on the late *p10* promoter in vitro and in vivo are shown in Figure 3. All *hr*s significantly increased the transcriptional activity of the *p10* promoter in both assays, with the exception of *hr4R* (0.84-fold in vitro, 1.05-fold in vivo). This activation ranged from 2.8- to 37.7-fold in BmN cells and from 7.3- to 79.2-fold in fifth-instar silkworm larvae. Overall, the enhancement of the *p10* promoter activities by *hr*s was greater in vivo than in vitro. Among the *hr*s, *hr*5 showed the strongest enhancer activity in both assays (37.7-fold in vitro, 79.2-fold in vivo).

### 3.5. Correlations Between Enhancer Efficiency and Numbers of Characteristic Sequences

The seven *hr*s of *Bm*NPV-YT showed significant enhancer activity on both homologous and heterologous promoters, and Spearman’s correlation analyses revealed that their enhancer efficiency was consistently associated with three key structural features: the number of palindromic repeats, the number of host bZIP (transcription-factor)-binding motifs (cAMP-response elements [CREs], and TPA-response elements [TREs]), and the total number of composite characteristic sequences.

For the homologous *ie1* promoter, enhancer efficiency showed highly significant positive correlations with (i) the number of palindromic sequences (r = 0.873, *p* = 0.010 < 0.05), (ii) the combined number of CRE and TRE motifs (r = 0.927, *p* = 0.003 < 0.01), and (iii) the total number of characteristic sequences (r = 0.964, *p* = 0.000 < 0.001).

Similarly, for the heterologous *ie1* promoter, enhancer efficiency correlated significantly with (i) the number of palindromic sequences (r = 0.927, *p* = 0.003 < 0.01), (ii) the combined number of CRE and TRE motifs (r = 0.873, *p* = 0.010 < 0.05), and (iii) the total number of characteristic sequences (r = 0.929, *p* = 0.003 < 0.001).

For the homologous *p10* promoter, enhancer efficiency also correlated strongly with (i) palindrome count (r = 0.982, *p* = 0.000 < 0.001), (ii) the combined number of CRE and TRE motifs (r = 0.901, *p* = 0.006 < 0.01), and (iii) the total number of characteristic sequences (r = 0.929, *p* = 0.003 < 0.001).

## 4. Discussion

Homologous regions (*hr*s) in baculoviruses are multifunctional *cis*-acting regulatory elements that play two critical roles: as origins of viral DNA replication and as potent transcriptional enhancers that orchestrate the spatiotemporal dynamics of viral gene expression throughout infection [38]. Widely conserved across lepidopteran nucleopolyhedroviruses, *hr*s are essential for the efficient replication of the viral genome, progeny virion production, and dynamic host–virus transcriptional crosstalk [14]. The majority of previous studies on baculoviral *hr*s have focused on characterizing individual *hr* elements, with functional analyses predominantly performed at the cellular level; yet the functional diversity and potential synergy among all *hr*s within a single viral strain have remained poorly explored. In this study, we systematically characterized the complete set of seven *hr*s from a newly isolated BmNPV-YT strain and profiled their enhancer activities toward homologous and heterologous immediate-early *ie1* promoters and the endogenous very-late *p10* promoter in two classic insect cell lines (BmN and Sf21 cells) and in silkworm larvae. To our knowledge, this represents the first comprehensive comparative analysis of all *hr*s from a signal BmNPV isolate conducted at both cellular and in vivo levels.

Our transient reporter assays confirmed that all seven *hr*s exerted intrinsic enhancer activity, consistent with previous reports that baculoviral *hr*s, particularly BmNPV-ZJ8 *hr*3, function as super-enhancers capable of elevating transcription by several orders of magnitude [12,32]. Importantly, the enhancer activities were evaluated under distinct physiological conditions: the *ie1* promoter assays were performed in non-virus-infected BmN and Sf21 cells and silkworm larvae, whereas *p10* promoter activity was determined in BmNPV-infected systems. Rather than displaying uniform functional redundancy, these BmNPV-YT *hr*s showed marked functional divergence. *hr*2L and *hr*3 showed extraordinarily high activation of the non-infected *ie1* promoter, whereas *hr*5 showed unique specificity for the virus-dependent very-late *p10* promoter. In striking contrast, *hr*4R showed substantially weaker enhancement of the *ie1* promoter relative to the other *hr*s and completely failed to activate the *p10* promoter in all the models tested.

Consistent with these functional observations, DNA pull-down combined with liquid chromatography–tandem mass spectrometry (LC–MS/MS) revealed that *hr*2L, *hr*3, and *hr*5, all of which displayed robust enhancer activity in our assays, are high-frequency interacting regions, together with *hr*1 [39]. A total of 215–612 specific binding proteins were identified for each of these four *hr*s [39]. These findings confirm that BmNPV *hr*s have evolved distinct regulatory capacities, despite their conserved repetitive structures. The defective activity of *hr*4R is probably attributable to its single repeat unit and a point mutation within the conserved *Eco*RI site, which may impair the binding of the host transcription factor and disrupt synergistic enhancer–protein interactions [40,41].

To explain the observed functional divergence among the *hr*s, we quantified multiple structural parameters and performed Spearman correlation analyses. Our results revealed that the enhancer potency of *hrs* from BmNPV-YT toward different promoters correlated significantly with the abundance of palindromic repeats, the combined abundance of CRE and TRE motifs (bZIP-binding sites), and the overall sequence composition. These findings are consistent with accumulated evidence that baculoviral *hr*s act as sophisticated *cis*-regulatory modules rather than simple repetitive sequences, in which palindromic sequences and interpalindromic CRE and TRE motifs act as core functional elements. These conserved motifs recruit host bZIP transcription factors to modulate Pol-II-dependent transcription and synergize with viral IE1 to maximize *hr* enhancer activity [42]. Moreover, such CRE/TRE architectural configurations are highly conserved across lepidopteran nucleopolyhedroviruses, reflecting the strong evolutionary selection pressure that sustains host–virus transcriptional crosstalk. Our quantitative correlation results provide preliminary support for a structure-dependent regulatory model governing the potency of *hr* enhancers, but given the limited sample size of seven *hr* elements, these observations should be validated by further experimentation.

All seven BmNPV-YT *hr*s exerted significant enhancer activity on the heterologous AcMNPV *ie1* promoter. However, their degrees of enhancement (13–449-fold) in silkworm larvae were consistently lower than those observed for the homologous BmNPV *ie1* promoter (43–1545-fold). This pattern echoes previous reports of BmNPV-ZJ8 *hr*3, which displayed significantly lower enhancer activity toward the heterologous *ie1* promoter than the homologous counterpart in silkworm larvae, with 605-fold and 1059-fold enhancement, respectively [12,24]. Such conserved cross-species regulatory compatibility indicates a universal mechanism underlying *hr*-mediated transcriptional activation across baculoviruses, whereas the pronounced in vivo regulatory discrepancy underscores the essential roles of host-specific cellular factors in modulating *hr* function. Silkworm larvae possess a unique nuclear microenvironment, enriched with co-evolved transcription factors and epigenetic regulators, which preferentially stabilize the functional enhancer complexes assembled with native BmNPV *hr*s [43]. Recent studies of chromatin interactions have revealed that BmNPV physically engages with host super-enhancer regions, hijacking transcription factors such as GATA to redirect cellular transcriptional resources toward viral gene expression—a regulatory paradigm optimized in its natural host [43]. In contrast, heterologous cellular systems lack these finely tuned molecular interactions. Therefore, although baculovirus *hr* elements display broad compatibility across different species and promoters, the realization of their full enhancer function is strictly environment-dependent: maximal enhancement is preferentially achieved when *hr*s match the homologous promoter and it occurs within the co-evolved host nuclear microenvironment [12,24,42].

The BmNPV *p10* gene is a very late gene encoding the P10 structural protein, which is essential for the morphogenesis of occlusion bodies and host cell lysis [44]. Consistent with our reporter assay results, all BmNPV-YT *hr*s except *hr*4R exerted measurable enhancement on the activity of the *p10* promoter. The differential activation of the early *ie1* and very-late *p10* promoters stems from intrinsic disparities in the promoter architecture and stage-specific transcriptional dependence. As an early-phase promoter, the *ie1* promoter relies heavily on *hr cis*-regulatory elements to recruit host Pol II and general transcription factors for robust transcriptional activation under noninfection conditions [6]. In contrast, *p10* is a canonical very-late viral gene, the transcription of which is strictly governed by virus-encoded RNA polymerase and the essential auxiliary factor VLF-1, a process distinct from host Pol-II-dependent early transcription [45,46]. Consequently, the *p10* promoter has a strong autonomous transcriptional capacity during the very-late stage of infection, rendering its *hr*-mediated enhancement auxiliary rather than essential. Notably, the seven *hr*s showed divergent enhancer efficiency rankings between the early and very-late promoters, indicating the distinct functional compatibility of different *hr*s with stage-specific viral and host transcription complexes. In particular, *hr*5 (the second largest of the seven *hr*s) displayed relatively weaker enhancement toward the *ie1* promoter than did *hr*2L or *hr*3, but had the strongest activating effect on the *p10* promoter of all the *hr*s tested, reflecting its unique functional preference for the very-late transcriptional machinery to drive the specific expression of the late viral genes [47,48]. Collectively, these findings indicate that baculoviruses use modular *hr* regulatory elements to dynamically coordinate the host and viral transcriptional machinery across distinct infection stages, allowing for the precise spatiotemporal control of viral gene expression throughout the infection cycle.

Despite these novel mechanistic insights into BmNPV *hr* functions, several limitations inherent to the present study should be acknowledged. First, all functional assessments relied on transient reporter systems, which cannot fully replicate the native viral infection context. Therefore, future work will employ recombinant baculoviruses to characterize the regulatory functions of *hr*s during authentic viral infection. Second, based on a correlational analysis of seven *hr* elements from the BmNPV-YT isolate, we observed strong correlations between structural features and *hr* enhancer activity. Nevertheless, owing to the limited sample size, these observed structure–function relationships should not be generalized to other baculoviruses. Moreover, future work will use targeted mutagenesis of palindromic units and core CRE and TRE motifs to directly verify the underlying causal relationships. To date, only the *hr*5-deleted mutant derived from BmNPV T3 has been characterized in previous research [33]; loss of *hr*5 exerted no influence on viral replication and infectivity, revealing that *hr*5 is non-essential for BmNPV propagation. To our knowledge, no studies have yet reported how the deletion of any single *hr* element affects viral transcriptional activity in BmNPV. Accordingly, further functional investigations via targeted knockout of single or multiple *hr*s in the BmNPV-YT genome are warranted to identify which *hr*s are indispensable for genome replication and transcriptional enhancement.

## 5. Conclusions

In this study, we systematically characterized seven *hr*s from the newly isolated BmNPV-YT strain using both cell-based assays and living silkworm larvae. These *hr*s displayed marked divergence in their functional activities, promoter preferences, host adaptations, and structural features. We performed the first systematic comparison of the complete set of seven *hr*s from a single BmNPV isolate in terms of their enhancer efficiency, promoter specificity (*ie1* versus *p10*), and host specificity (BmN cells, Sf21 cells, and silkworm larvae), and identified quantitative correlations linking palindromic repeat abundance, CRE/TRE motif content, and total characteristic sequence count to *hr* regulatory strength. From an applied perspective, the highly efficient enhancers *hr*2L and *hr*3 identified here can be combined with early promoters to significantly enhance the expression of foreign proteins in the BEVS, providing optimized *cis*-regulatory tools for viral-vector-based biomanufacturing. Collectively, this work advances our fundamental understanding of baculoviral spatiotemporal transcriptional regulation and offers practical tools for the development of next-generation BEVS platforms.

## Figures and Tables

**Table 1:** Primers used for construction of reporter plasmids.

Primer	Sequence (5′–3′)
Bm-*hr*1-F	CGGGATCCCAATGACATCATCGTTTGATTG (*BamH*I)
Bm-*hr*1-R	CGGTCGACTATATACTTGCTTTACGAGTAG (*Sal*I)
Bm-*hr*2L-F	CGGGATCCATATGTAAATGATGTCATCG (*BamH*I)
Bm-*hr*2L-R	CGGTCGACGACAGCAT CCACTGATCTTG (*Sal*I)
Bm-*hr*2R-F	CGGGATCCTAAATGACATCATCCCTTGA (*BamH*I)
Bm-*hr*2R-R	CGGTCGACCAAATTATTAACTAGTCTCA (*Sal*I)
Bm-*hr*3-F	CGGGATCCTGACATCATTCCTGATTATA (*BamH*I)
Bm-*hr*3-R	CGGTCGACTAACTAAACTCGCTTTACGA (*Sal*I)
Bm-*hr*4L-F	CGGGATCCTATGACTCATCAATCGATCG (*BamH*I)
Bm-*hr*4L-R	CGGTCGACCGTTGTTTCATTGACGTCAC (*Sal*I)
Bm-*hr*4R-F	CGGGATCCTATAATCAAGAAATGATGTC (*BamH*I)
Bm-*hr*4R-R	CGGTCGACCTGTGATCGTGTTTTACACG (*Sal*I)
Bm-*hr*5-F	CGGGATCCGCAAGATTTGAGATGATGTC (*BamH*I)
Bm-*hr*5-R	CGGTCGACCGTGTGCAAAACATGACATC (*Sal*I)
p3-Bm-*p10*P-F	GAGGTACCATGTCGGGACACATAAAGGT (*Kpn*I)
p3-Bm-*p10*P-R	CGAAGCTTGCGTAATTTACAGTATAAG (*Hind*III)

Note: Restriction enzyme sites are underlined.

**Table 2:** Number and types of sequence motifs found within seven *hr*s of BmNPV-YT.

Motif	*hr*1	*hr*2L	*hr*2R	*hr*3	*hr*4L	*hr*4R	*hr*5
Imperfect palindromes	5	7	4	6	5	1	7
Stem–loop structure	4	0	6	6	5	0	0
CGATT motif	0	1	0	1	2	2	0
MLTF/USF	3	10	6	6	7	6	9
CRE motif	5	13	8	9	7	2	12
TRE motif	0	1	0	0	1	2	1
Sum of motifs	17	32	24	28	27	13	29

Note: Imperfect palindromes, GTTTTACAAGTAGAATTCTACTCGTAAAGC; Stem–loop structure, TTTGAAAAACAAA; MLTF/USF, CANNTG; CRE motifs, TGA(TG/CA)TCA; TRE motifs, TGACTCA.

**Table 3:** Seven *hr*s of BmNPV-YT enhanced homologous *ie1* promoter activity in BmN.

Reporter Plasmids	CPM	Stimulation Folds
pBm*ie1*P/luc-*hr*1	1.05 × 10^7^ ± 2.33 × 10^5^	27
pBm*ie1*P/luc-*hr*2L	4.82 × 10^8^ ± 5.10 × 10^6^	1258
pBm*ie1*P/luc-*hr*2R	2.17 × 10^7^ ± 2.93 × 10^4^	57
pBm*ie1*P/luc-*hr*3	2.76 × 10^8^ ± 5.51 × 10^5^	721
pBm*ie1*P/luc-*hr*4L	6.94 × 10^7^ ± 4.07 × 10^5^	181
pBm*ie1*P/luc-*hr*4R	4.29 × 10^6^ ± 7.62 × 10^4^	11
pBm*ie1*P/luc-*hr*5	1.81 × 10^8^ ± 3.92 × 10^5^	473
pBm*ie1*P/luc	3.83 × 10^5^ ± 2.98 × 10^3^	1
pGl3-Basic	35 ± 3	—

Note: The CPM value represented 50 µg of protein extracted from transfected BmN cells. Enhancing ability of seven *hr*s of BmNPV-YT inserted into the plasmids pBm*ie1*P/luc-*hr*1, pBm*ie1*P/luc-*hr*2L, pBm*ie1*P/luc-*hr*2R, pBm*ie1*P/luc-*hr*3, pBm*ie1*P/luc-*hr*4L, pBm*ie1*P/luc-*hr*4R, and pBm*ie1*P/luc-*hr*5 were expressed as stimulation folds relative to the plasmid pBm*ie1*P/luc+, which was arbitrarily set as 1. The plasmid pGl3-Basic was used as the basic control. Results are presented as means ± SD from triplicate assays across three independent experiments.

**Table 4:** Seven *hr*s of BmNPV-YT enhanced homologous *ie1* promoter activity in silkworm larvae.

Reporter Plasmids	CPM	Stimulation Folds
pBm*ie1*P/luc-*hr*1	3.25 × 10^6^ ± 1.11 × 10^3^	71
pBm*ie1*P/luc-*hr*2L	7.08 × 10^7^ ± 7.41 × 10^5^	1545
pBm*ie1*P/luc-*hr*2R	6.73 × 10^6^ ± 4.06 × 10^3^	147
pBm*ie1*P/luc-*hr*3	4.70 × 10^7^ ± 2.37 × 10^5^	1026
pBm*ie1*P/luc-*hr*4L	1.25 × 10^7^ ± 3.85 × 10^4^	273
pBm*ie1*P/luc-*hr*4R	1.98 × 10^6^ ± 6.19 × 10^3^	43
pBm*ie1*P/luc-*hr*5	3.19 × 10^7^ ± 8.20 × 10^4^	696
pBm*ie1*P/luc	4.58 × 10^4^ ± 2.18 × 10^3^	1
pGl3-Basic	26 ± 2	—

Note: The CPM value represented 50 µg of protein extracted from transfected silkworm larvae. Enhancing ability of seven *hr*s of BmNPV-YT inserted into the plasmids pBm*ie1*P/luc-*hr*1, pBm*ie1*P/luc-*hr*2L, pBm*ie1*P/luc-*hr*2R, pBm*ie1*P/luc-*hr*3, pBm*ie1*P/luc-*hr*4L, pBm*ie1*P/luc-*hr*4R, and pBm*ie1*P/luc-*hr*5 were expressed as stimulation folds relative to the plasmid pBm*ie1*P/luc+, which was arbitrarily set as 1. The plasmid pGl3-Basic was used as the basic control. Results are presented as means ± SD from triplicate assays across three independent experiments.

**Table 5:** Seven *hr*s of BmNPV-YT enhanced heterologous *ie1* promoter activity in Sf21.

Reporter Plasmids	CPM	Stimulation Folds
pAc*ie1*P/luc-*hr*1	3.10 × 10^7^ ± 2.12 × 10^6^	57
pAc*ie1*P/luc-*hr*2L	5.58 × 10^8^ ± 6.79 × 10^6^	1023
pAc*ie1*P/luc-*hr*2R	1.65 × 10^7^ ± 1.99 × 10^5^	30
pAc*ie1*P/luc-*hr*3	5.06 × 10^8^ ± 3.66 × 10^5^	928
pAc*ie1*P/luc-*hr*4L	1.14 × 10^8^ ± 5.07 × 10^5^	209
pAc*ie1*P/luc-*hr*4R	1.03 × 10^7^ ± 2.93 × 10^5^	19
pAc*ie1*P/luc-*hr*5	2.17 × 10^8^ ± 4.22 × 10^5^	398
pAc*ie1*P/luc	5.45 × 10^5^ ± 3.29 × 10^3^	1
pGl3-Basic	24 ± 3	—

Note: The CPM value represented 50 µg of protein extracted from transfected Sf-21 cells. Enhancing ability of seven *hr*s of BmNPV-YT inserted into the plasmids pAc*ie1*P/luc-*hr*1, pAc*ie1*P/luc-*hr*2L, pAc*ie1*P/luc-*hr*2R, pAc*ie1*P/luc-*hr*3, pAc*ie1*P/luc-*hr*4L, pAc*ie1*P/luc-*hr*4R, and pAc*ie1*P/luc-*hr*5 were expressed as stimulation folds relative to the plasmid pAc*ie1*P/luc+, which was arbitrarily set as 1. The plasmid pGl3-Basic was used as the basic control. Results are presented as means ± SD from triplicate assays across three independent experiments.

**Table 6:** Seven *hr*s of BmNPV-YT enhanced heterologous *ie1* promoter activity in silkworm larvae.

Reporter Plasmids	CPM	Stimulation Folds
pAc*ie1*P/luc-*hr*1	2.50 × 10^6^ ± 5.92 × 10^4^	46
pAc*ie1*P/luc-*hr*2L	2.42 × 10^7^ ± 8.40 × 10^5^	449
pAc*ie1*P/luc-*hr*2R	2.04 × 10^6^ ± 3.79 × 10^5^	38
pAc*ie1*P/luc-*hr*3	1.87 × 10^7^ ± 1.11 × 10^6^	347
pAc*ie1*P/luc-*hr*4L	1.95 × 10^6^ ± 7.66 × 10^4^	36
pAc*ie1*P/luc-*hr*4R	7.02 × 10^5^ ± 1.62 × 10^4^	13
pAc*ie1*P/luc-*hr*5	1.13 × 10^7^ ± 1.91 × 10^6^	210
pAc*ie1*P/luc	5.39 × 10^4^ ± 2.31 × 10^3^	1
pGl3-Basic	18 ± 2	—

Note: The CPM value represented 50 µg of protein extracted from transfected silkworm larvae. Enhancing ability of seven *hr*s of BmNPV-YT inserted into the plasmids pAc*ie1*P/luc-*hr*1, pAc*ie1*P/luc-*hr*2L, pAc*ie1*P/luc-*hr*2R, pAc*ie1*P/luc-*hr*3, pAc*ie1*P/luc-*hr*4L, pAc*ie1*P/luc-*hr*4R, and pAc*ie1*P/luc-*hr*5 were expressed as stimulation folds relative to the plasmid pAc*ie1*P/luc+, which was arbitrarily set as 1. The plasmid pGl3-Basic was used as the basic control. Results are presented as means ± SD from triplicate assays across three independent experiments.

## Data Availability

The original contributions presented in this study are included in the article. Further inquiries can be directed to the corresponding author.
